# Class I-Histone Deacetylase (HDAC) Inhibition is Superior to pan-HDAC Inhibition in Modulating Cisplatin Potency in High Grade Serous Ovarian Cancer Cell Lines

**DOI:** 10.3390/ijms20123052

**Published:** 2019-06-22

**Authors:** Jan J. Bandolik, Alexandra Hamacher, Christian Schrenk, Robin Weishaupt, Matthias U. Kassack

**Affiliations:** 1 Institute for Pharmaceutical and Medicinal Chemistry, University of Duesseldorf, 40225 Duesseldorf, Germany; jan.bandolik@hhu.de (J.J.B.); alexandra.hamacher@hhu.de (A.H.); schrenc@hhu.de (C.S.); 2 Institute for Computer Science, Computational Complexity and Cryptology, University of Duesseldorf, 40225 Duesseldorf, Germany; robin.weishaupt@hhu.de

**Keywords:** cisplatin, high grade serous ovarian cancer (HGSOC), histone deacetylase inhibitors, caspase activity, antitumor platinum agents, combination treatment, panobinostat, entinostat, nexturastat A

## Abstract

High grade serous ovarian cancer (HGSOC) is the most common and aggressive ovarian cancer subtype with the worst clinical outcome due to intrinsic or acquired drug resistance. Standard treatment involves platinum compounds. Cancer development and chemoresistance is often associated with an increase in histone deacetylase (HDAC) activity. The purpose of this study was to examine the potential of HDAC inhibitors (HDACi) to increase platinum potency in HGSOC. Four HGSOC cell lines with different cisplatin sensitivity were treated with combinations of cisplatin and entinostat (class I HDACi), panobinostat (pan-HDACi), or nexturastat A (class IIb HDACi), respectively. Inhibition of class I HDACs by entinostat turned out superior in increasing cisplatin potency than pan-HDAC inhibition in cell viability assays (MTT), apoptosis induction (subG1), and caspase 3/7 activation. Entinostat was synergistic with cisplatin in all cell lines in MTT and caspase activation assays. MTT assays gave combination indices (CI values) < 0.9 indicating synergism. The effect of HDAC inhibitors could be attributed to the upregulation of pro-apoptotic genes (*CDNK1A*, *APAF1*, *PUMA*, *BAK1*) and downregulation of *survivin*. In conclusion, the combination of entinostat and cisplatin is synergistic in HGSOC and could be an effective strategy for the treatment of aggressive ovarian cancer.

## 1. Introduction

Ovarian cancer is one of the most lethal gynecological cancer subtypes, such as uterus, breast, cervical or vulva cancer. Compared to the other gynecological cancers, ovarian cancers have the worst survival rate over five years (47.6%, USA; 41%, Germany). In contrast, breast cancer has a 5 year survival rate of 89.9% in the USA and 88% in Germany [1,2,3,4,5,6]. Ovarian cancer can be divided into types I and II. Type I is usually less aggressive and contains the subtypes endometrioid carcinoma and clear cell carcinoma as well as mucinous carcinoma and low-grade serous carcinoma. However, cohort studies from the USA found out that 68% of ovarian cancer cases are type II diseases [7,8]. Type II ovarian cancer is clinically very aggressive and consists of high grade serous ovarian cancer (HGSOC). HGSOC is rapidly growing, shows genomic instability, and dysfunction in tumor suppressors [9,10]. Primary options for treatment are surgery and a combination chemotherapy including platinum compounds like cisplatin or carboplatin and paclitaxel [11]. Initially, most ovarian cancers are chemosensitive. During the treatment, resistance develops and leads to relapse and therapeutic failure. The mechanisms behind a developing platinum resistance are multifactorial in nature and cannot be overcome by increasing the dose of a platinum drug given to a patient [12]. Furthermore, with increasing doses, patients suffer from increased frequency or intensity of side effects like nephrotoxicity or ototoxicity. Combination therapies with small molecule inhibitors are a widely used strategy to increase the platinum sensitivity of the tumor or to prevent/delay the development of therapeutic resistance [13,14]. It is well known that epigenetic modifications such as histone acetylation or DNA methylation are dysregulated in cancer and moreover in chemoresistance development [15,16]. The acetylation and deacetylation of proteins is regulated by histone acetyl transferases (HATs) and histone deacetylase (HDAC) enzymes. Eleven human zinc-dependent HDACs are known (HDAC1-11) which can be clustered into several classes: Class I: HDAC1, 2, 3, 8; class IIa: HDAC4, 5, 7, 9; class IIb: HDAC6, 10; and class IV: HDAC11. Class III comprises non-zinc-dependent HDAC enzymes, the so-called sirtuines which will not be considered in this paper [17]. There are several known overexpression patterns of histone deacetylase (HDAC) enzymes in different cancer subtypes leading to transcriptional repression [15]. For ovarian cancer, an overexpression of HDACs - especially of class I HDACs - is described and correlates with its aggressiveness. Furthermore, HDAC overexpression can serve as a marker of poor clinical outcome [18]. It is also described that chemotherapy leads to an upregulation of HDAC1 expression [19].

Thus, using HDAC inhibitors (HDACi) to normalize a dysregulated acetylation status of histone and non-histone proteins is an increasingly pursued strategy to increase the sensitivity of chemotherapeutic agents [20,21,22]. HDACi can act by regulating pro- and anti-apoptotic gene expression and thus promoting cell cycle arrest and induction of apoptosis [16]. However, the exact regulatory network of HDACi on apoptosis induction in a particular type of cancer is not fully understood. Further, it is not yet clear if subtype-selective HDACi or pan-HDAC inhibition is superior in increasing chemosensitivity of anticancer drugs such as platinum compounds.

The purpose of this study was to test the effect of HDACi with different subtype preferences on the chemosensitivity of cisplatin in HGSOC cell lines. Panobinostat was used as pan-HDACi, entinostat as class I selective HDACi, and nexturastat A as class IIb (HDAC6)-selective HDACi. Due to the intrinsic effect of HDACi regulating gene expression, all inhibitors were incubated 48 h prior to cisplatin treatment. CaOV3, Kuramochi, OVSAHO, and HEY cell lines were chosen as representatives for HGSOC based on literature recommendations [23,24,25,26]. Among these HGSOC cell lines, CaOV3, Kuramochi, and OVSAHO are described as very suitable cellular models for HGSOC [27]. In addition to the four HGSOC cell lines, A2780 was included as a comparison model for ovarian endometrioid adenocarcinoma (type I) [28,29]—a less aggressive type of ovarian cancer. HDACi—induced effects on cisplatin sensitivity were investigated using cell viability assays, apoptosis induction, caspase activation, and analysis of apoptosis-related gene expression. Notably, the class I HDACi entinostat turned out superior to panobinostat in increasing cisplatin potency in HGSOC and was synergistic with cisplatin. The chemosensitizing effect could be attributed to the upregulation of cell cycle arrest and pro-apoptotic genes.

## 2. Results

### 2.1. Characterization of the Human Ovarian Cancer Cell Lines

**Figure 1:**
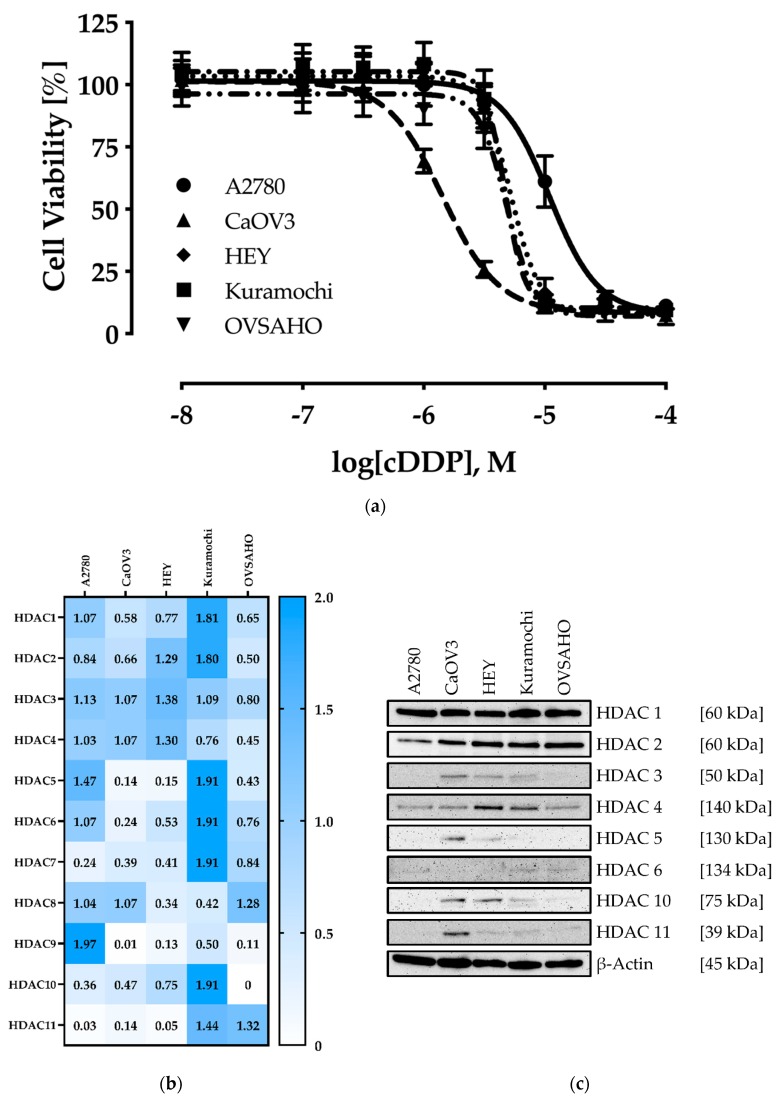
Characterization of the ovarian cancer cell lines. (**a**) Cell viability was measured by MTT assay after 72 h incubation with different concentrations of cisplatin. Data shown are mean ± SD of at least three independent experiments each carried out in triplicates, calculated as percentage of control. The cell line specific IC_50_ values are shown in Table 1. (**b**) Gene expression profile for HDAC enzymes was obtained by RT-PCR. Data shown are normalized to endogenous control gene expression of *HPRT1* (hypoxanthine-guanine phosphoribosyltransferase), *TBP* (TATA binding protein), and *GUSB* (beta-glucuronidase). (**c**) Representative immunoblot analysis of HDAC enzymes. One representative immunoblot with a protein molecular weight marker is shown in Figure S3.

We chose the human ovarian cancer cell lines A2780, CaOV3, HEY, Kuramochi, and OVSAHO as cellular models for HGSOC (CaOV3, HEY, Kuramochi, and OVSAHO) and ovarian endometrioid adenocarcinoma type I (A2780). First, the cisplatin sensitivity of the cell lines and their HDAC expression profile (mRNA, protein) was analyzed. The results are shown in Figure 1.

IC_50_ values of the cell lines towards cisplatin ranged from 1.44 µM for CaOV3 up to 11.0 µM for A2780 (Figure 1A and Table 1). In literature, cisplatin plasma levels of 1.90 to 8.72 µM are reported in patients (c_max_ reached after 1–1.5 h) [30].

A2780 cells showed IC_50_-values for cisplatin in the one-digit micromolar range in previous studies [20,21,31]. Nevertheless, under the experimental conditions chosen here, an IC_50_-value of 11.0 µM was obtained. Based on the IC_50_-values, CaOV3 cells can be classified as cisplatin-sensitive, while HEY, Kuramochi, and OVSAHO cells are mediocre sensitive and A2780 cisplatin-resistant.

Regarding the HDAC enzyme expression, different patterns were obtained. At mRNA level, the cell line Kuramochi showed the highest expression of *HDAC 1*, *2*, *5*, *6*, *7*, *10*, and *11* whereas A2780 revealed the highest expression of *HDAC9*. CaOV3, HEY, and OVSAHO generally showed a low expression of HDACs in comparison to Kuramochi (Figure 1B). Within a particular cell line, *HDAC1-4* showed a rather high expression except for Kuramochi and A2780.

Comparing the HDAC gene expression profile with the HDAC protein expression levels, some differences become evident (Figure 1C). HDAC1 and HDAC2 are well expressed in every cell line, whereas HDAC3 was only detected (protein) in CAOV, HEY, and Kuramochi. HDAC4 (class IIa HDAC) is expressed in every cell line with a slightly higher expression in HEY and Kuramochi. HDAC5 is only expressed in CaOV3 and slightly in HEY cells. For HDAC6 and HDAC10, members of class IIb, a more heterogenous expression profile occurred. While HDAC10 is strongly expressed in CaOV3 and HEY cells, it is weakly expressed especially in Kuramochi. For HDAC6 we observed a slight expression in A2780, Kuramochi, and OVSAHO. Expression levels for HDAC11 as representative of class III HDACs show some differences, too. CaOV3 expresses HDAC11 at a higher level, HEY, Kuramochi, and OVSAHO at a very weak level, and A2780 shows no expression. For HDAC4 and HDAC6 a good correlation between mRNA and protein expression levels could be observed. In conclusion, class I HDACs HDAC1 and HDAC2 were highly expressed throughout all ovarian cancer cell lines whereas the other HDAC isoforms are expressed at a much lower level and only in some but not all cell lines (Figure 1B,C).

### 2.2. Cytotoxic and HDAC-Inhibitory Effects of Entinostat, Panobinostat, and Nexturastat A

Next, we analyzed the antiproliferative effects of entinostat, panobinostat, and nexturastat A in the ovarian cancer cell lines. Incubation times applied for the investigation of HDACi alone (absence of cisplatin) were the same as later (chapter 2.3) used in combination experiments of HDACi with cisplatin. Results are shown in Table 2.

Panobinostat showed the highest cytotoxic effects against all five cell lines with IC_50_-values in the low nanomolar range. Entinostat had moderate cytotoxicity with IC_50_-values in the submicromolar range. Nexturastat A had micromolar IC_50_-values. Thus, nexturastat A was not cytotoxic at concentrations where it displays HDAC6 selectivity (in the nanomolar range). To determine the HDAC inhibitory activity of entinostat, panobinostat, and nexturastat A, we performed a whole-cell HDAC inhibition assay. Results are shown in Table 3.

**Figure 2:**
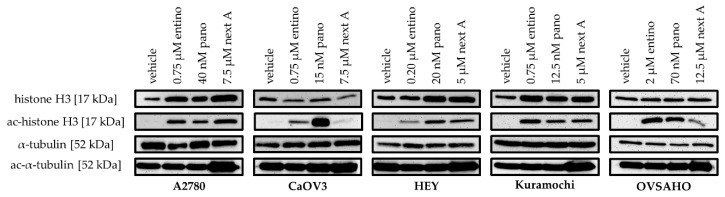
Effect of HDACi on acetylation level of α-tubulin and histone H3. Representative immunoblot analysis of histone H3, ac-histone H3, α-tubulin, and ac-α-tubulin in the different human ovarian cancer cell lines. Cells were treated with the indicated concentrations of HDACi. Control cells were incubated with vehicle. One representative immunoblot with a protein molecular weight marker is shown in Figure S3.

No significant differences were observed between the cell lines regarding the HDAC inhibitory potency of entinostat. The IC_50_-values for entinostat ranged from 0.22 µM in HEY cells to 0.34 µM in Kuramochi cells. As expected, panobinostat showed IC_50_-values in the nanomolar range. The IC_50_-values were between 7.88 nM for CaOV3 cells and 23.1 nM for OVSAHO cells. For nexturastat A, we obtained IC_50_-values in the low micromolar range and could not detect a large difference in the IC_50_-values between the individual cell lines. The IC_50_-values ranged from 3.63 µM for A2780 cells to 5.25 µM in OVSAHO cells. For confirmation of HDAC-inhibitory effects, α-tubulin and histone H3 acetylation were analyzed by western blot (Figure 2).

As can be seen, treatment with the HDACi was associated with a hyperacetylation of histone H3 indicating class I HDAC inhibitory effects. Hyperacetylation of α-tubulin is a marker for HDAC6 inhibition. Incubation with nexturastat A confirmed HDAC6 inhibition in every cell line by yielding increased levels of ac-α-tubulin.

### 2.3. Enhancement of Cisplatin-Induced Cytotoxicity

To investigate a possible effect of HDACi on the cisplatin sensitivity, the ovarian cancer cells were pretreated with HDACi for 48 h, respectively. After medium exchange, a 72 h treatment followed with freshly added HDACi and different concentrations of cisplatin. The concentrations of HDACi used in this combination experiment were adjusted to exert a maximum effect of 44–60% reduction of cell viability in the absence of cisplatin (i.e., a maximum reduction of the upper plateau of concentration-effect curves of 44–60%). Concentrations of HDACi used in combination treatments are shown in Table 4.

Whereas entinostat retained its class I-selective properties in the used concentration range, nexturastat A was likely to lose HDAC6 selectivity at 5–12.5 µM concentrations used in combinations with cisplatin. However, lower concentrations of nexturastat A did not have effects on the cisplatin sensitivity. To confirm the loss of selectivity of nexturastat A at high concentrations, we performed enzyme HDAC-inhibition assays with nexturastat on representative HDACs for class I, IIa, and IIb.

As can be seen in Table 5, nexturastat A is a nanomolar potency inhibitor at HDAC6 whereas the IC_50_-value at HDAC2 is 1.99 µM. However, using nexturastat A in combination experiments with cisplatin in concentrations ranging from 5 to 12.5 µM (Table 4) is highly likely to lead to a loss of HDAC6 selectivity. Thus, nexturastat A effects may be attributed to class I and HDAC6 inhibition rather than HDAC6 inhibition only.

**Figure 3:**
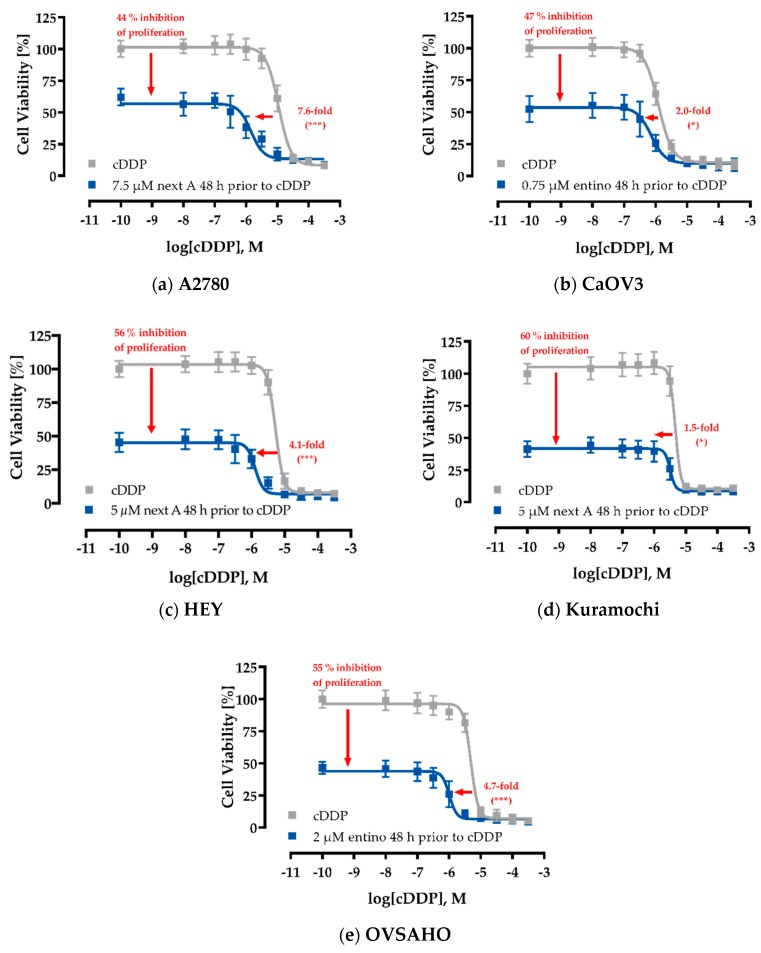
HDACi pretreatment enhances the cytotoxic effects of cisplatin. A2780 (**a**), CaOV3 (**b**), HEY (**c**), Kuramochi (**d**), and OVSAHO (**e**) were pretreated with the indicated HDACi 48 h prior to cisplatin (cDDP) administration. After another 72 h, IC_50_-values were determined by MTT assay. Data shown are normalized to vehicle control and mean ± SD of at least three experiments each carried out in triplicates. The vertical arrows show the antiproliferative effects of HDACi in the absence of cisplatin and the horizontal arrows show the shifts of the IC_50_-values of cisplatin (absence and presence of HDACi). Statistical analysis was performed using *t*-test. Levels of significance: ns (*p* > 0.05); * (*p* ≤ 0.05); ** (*p* ≤ 0.01); *** (*p* ≤ 0.001).

Table 6 summarizes the effects of 48 h pretreatment of HDACi on the cisplatin concentration-effect curves in all ovarian cancer cell lines. Besides IC_50_-values of cisplatin alone, IC_50_-values of cisplatin in the combination with the respective HDACi are displayed. Further, shift factors (ratio of IC_50_-value of cisplatin and the IC_50_-value of the combination) were calculated and included in Table 6. Figure 3 shows the corresponding concentration-effect curves of those HDACi–cisplatin combinations that achieved the largest effect in each of the cell lines.

Only class I-selective entinostat was able to increase cisplatin potency in each cell line in a significant manner (Table 6). Even in cisplatin-sensitive CaOV3 cells, entinostat was able to induce a slight, but significant enhancement of cisplatin potency (shift factor of 2.0, Figure 3B, Table 6). Within the HGSOC cell lines, entinostat gave the highest shift factors (except for HEY and Kuramochi cells where shift factors of entinostat and nexturastat A were not significantly different from each other). Entinostat showed the strongest effect in OVSAHO cells (SF = 4.7, Table 6, Figure 3E). Notably, a similar shift factor was achieved in HEY cells (SF = 3.8) with a remarkably low pretreatment-concentration of entinostat (200 nM) whereas the other cell lines were treated with 750 nM (A2780, CAOV, Kuramochi) or even 2000 nM (OVSAHO) entinostat. Surprisingly, the pan HDACi panobinostat was not superior but even less efficacious than entinostat to increase cisplatin potency except in A2780 cells. However, A2780 do not belong to HGSOC. Nexturastat A showed similar efficacy as entinostat. However, it needs to be considered that the concentrations used for nexturastat A (5 to 12.5 µM) lead to a loss of HDAC6 selectivity. Almost complete inhibition of class I HDACs can be assumed considering the K_i_ values presented in Table 5. Reducing nexturastat A concentration in pretreatment to 2.5 µM led to complete loss of chemosensitizing activity in HEY cells (Figure S2).

To analyze the type of interaction between HDACi and cisplatin, synergism studies according to Chou-Talalay were performed using MTT assay [32,33]. The cell lines were incubated with varying concentrations of HDACi and cisplatin. Cells were pretreated for 48 h with HDACi alone followed by 72 h treatment with HDACi and cisplatin (termed: “48 h + 72 h”). Concentrations were chosen to achieve a fraction affected (f_a_; level of cell viability inhibition) between 0.2 and 0.9 in the Chou-Talalay-analysis. Combination indices (CI) were calculated and presented in Table 7.

The interactions between cisplatin and entinostat, panobinostat, or nexturastat A, respectively, were synergistic as indicated by CI-values lower than 0.9. Most CI values were lower than 0.5 indicating strong synergism.

### 2.4. Enhancement of Cisplatin-Induced Cytotoxicity is Mediated via Apoptosis-Induction

**Figure 4:**
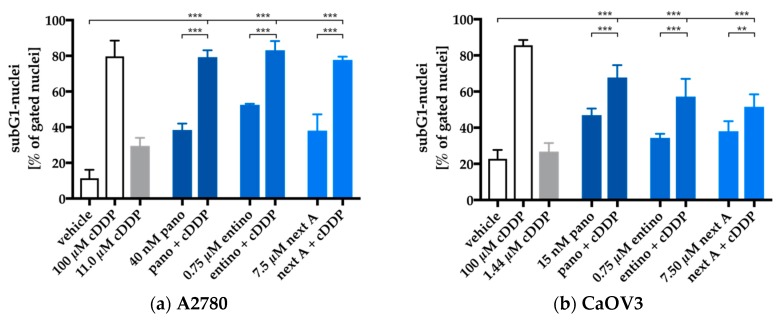

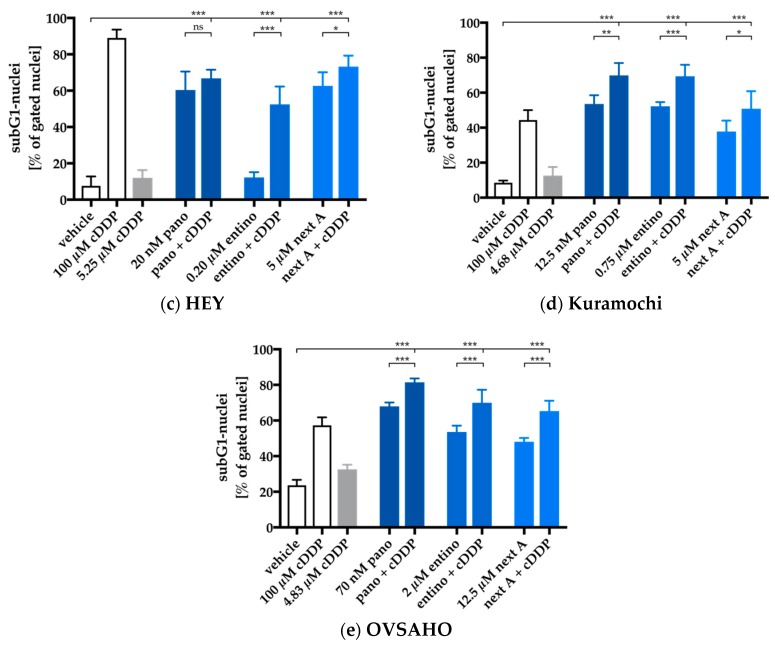
HDACi pretreatment enhances cisplatin-induced apoptosis. A2780 (**a**), CaOV3 (**b**), HEY (**c**), Kuramochi (**d**), and OVSAHO (**e**) cells were preincubated with HDACi for 48 h. Cisplatin was added in an IC_50_ concentration for each cell line for a further incubation period of 24 h. Apoptosis was analyzed by determining the sub-G1 cell fractions by flow cytometry analysis. 100 µM cisplatin served as positive control for apoptosis induction. 0.2% DMSO was added as a control for vehicle treated cells. All experimental conditions were incubated for same time periods. Data are the mean ± SD, n ≥ 2. Statistical analysis to compare the apoptosis induction by cisplatin or HDACi alone and the combination of HDACi and cisplatin was performed using *t*-test. Levels of significance: ns (*p* > 0.05); * (*p* ≤ 0.05); ** (*p* ≤ 0.01); *** (*p* ≤ 0.001).

Next, we investigated if the observed synergistic cytotoxic effect was mediated via apoptosis-induction. The cell lines were preincubated for 48 h with the same HDACi concentrations used for the MTT combination treatments (Figure 3, Table 6) followed by addition of cisplatin for another 24 h in an IC_50_ concentration. Then, cells were subjected to flow-cytometry-based subG1 analysis. Results are shown in Figure 4.

**Figure 5:**
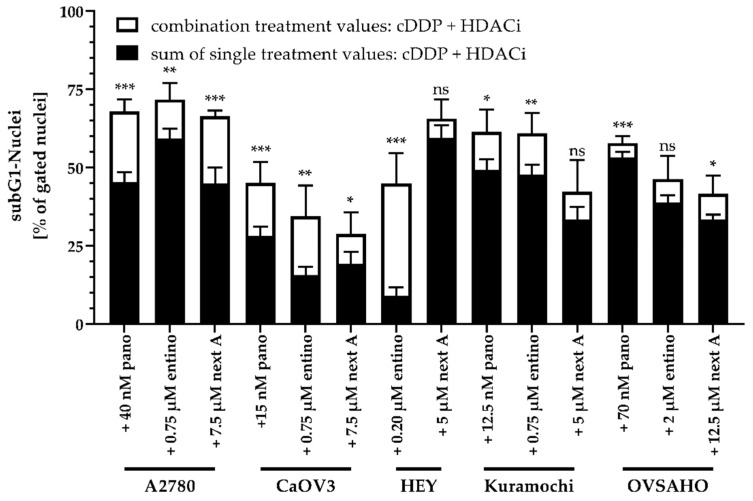
Synergistic effects of the combination of HDACi and cisplatin on apoptosis induction. The sum of single treatment effects (HDACi, cisplatin) are shown in black bars. The extended white bars show the difference (superadditive (= synergistic) part) between the sum of single treatment effects and the effect of combination treatments. Vehicle treated control was subtracted. Data are the mean ± SD. Statistical analysis was performed using *t*-test. Levels of significance: ns (*p* > 0.05); * (*p* ≤ 0.05); ** (*p* ≤ 0.01); *** (*p* ≤ 0.001).

Single treatment with each HDACi had an equal or higher effect on apoptosis induction than treatment with cisplatin alone in each cell line. Every combination treatment showed a significant increase in apoptosis induction compared to cisplatin or HDACi alone. Only the combination of cisplatin and panobinostat in HEY cells showed no difference in apoptosis induction, possibly due to the unexpected high apoptotic effect of panobinostat alone. In order to determine whether the effects of HDACi and cisplatin combinations on apoptosis induction were hyperadditive (= synergistic), we compared the sum of the single treatment effects of HDACi and cisplatin (subG1 nuclei) with the results from the combination treatment (Figure 5). Data were only analyzed if there was a significant increase in subG1 induction in Figure 4.

Interestingly, most combinations turned out to be synergistic. Only nexturastat A in HEY and Kuramochi and entinostat in OVSAHO showed no significant difference, probably due to the larger error of the entinostat data. Entinostat showed the strongest increase in subG1 nuclei in HEY cells. Overall, these combination treatment data from apoptosis induction confirm MTT data (Table 6 and Table 7).

### 2.5. Apoptosis-Induction of the Combination Treatment is Caspase3/7-Driven

**Figure 6:**
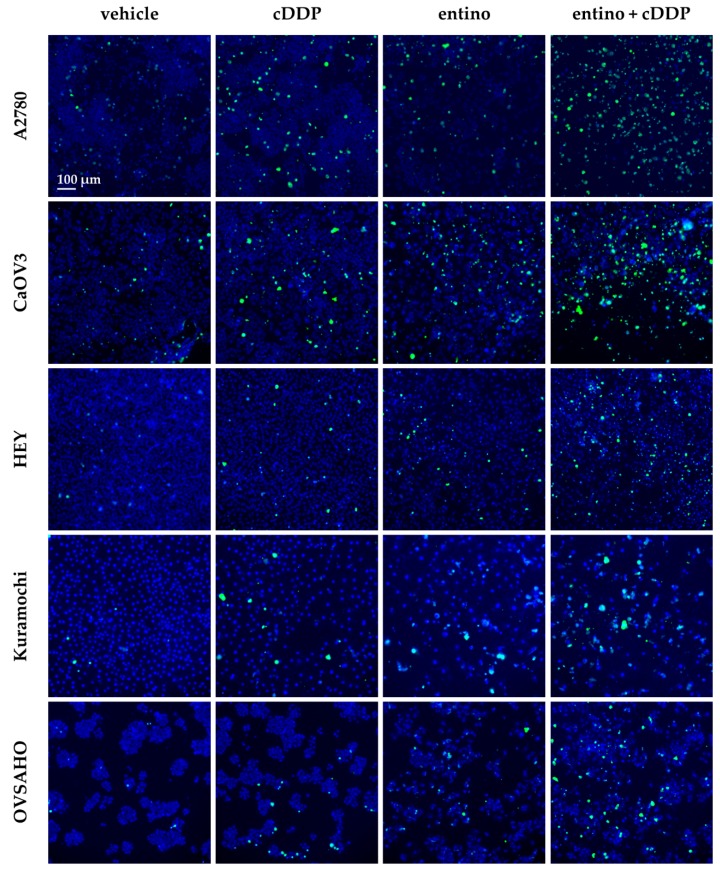
Representative fluorescent imaging pictures (10× magnification) are shown for each cell line for the treatment of cisplatin (IC_50_ concentration), entinostat, and the combination of cisplatin and entinostat. Cell nuclei were stained by Hoechst 33342 and appear blue while cells with activated caspases3/7 showed green fluorescence. Scale bar in upper left image is 100 µm and applies to all images.

Here, we tested if apoptosis induction is caspase dependent. Caspase3/7-activation was measured by fluorescence imaging. Untreated controls, single compound and combined treatments of HDACi and cisplatin were analyzed. Figure 6 shows as an example fluorescence images for untreated control (vehicle), cisplatin, entinostat, and the combination of entinostat and cisplatin for each cell line.

**Figure 7:**
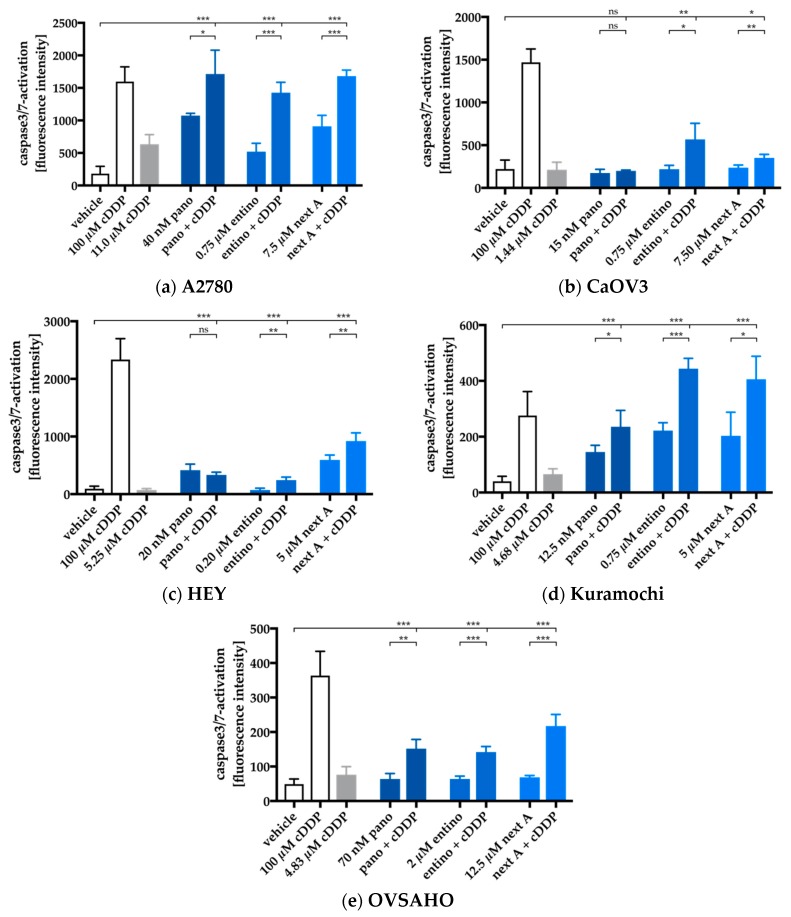
A2780 (**a**), CaOV3 (**b**), HEY (**c**), Kuramochi (**d**), and OVSAHO (**e**) cells were preincubated with HDACi for 48 h. Cisplatin was added in an IC_50_ concentration for each cell line for a further incubation period of 24 h. Caspase3/7-activation was analyzed by incubation with CellEvent Caspase-3/7 green detection reagent (Thermo Scientific, Germany) and visualized by ArrayScan XTI. Cisplatin 100 µM (24 h) was added as positive control for caspase3/7-activation. 0.2% DMSO was added as a control for vehicle treated cells. All experimental conditions were incubated for same time periods. To verify the involvement of caspases in the observed effects, 20 µM QVD was preincubated for 30 min prior to compound addition. No caspase3/7-activation was obtained (data not shown). Data are the mean ± SD. Statistical analysis to compare the caspase3/7-activation by cisplatin or HDACi alone and the combination of HDACi and cisplatin was performed using *t*-test. Levels of significance: ns (*p* > 0.05); * (*p* ≤ 0.05); ** (*p* ≤ 0.01); *** (*p* ≤ 0.001).

Caspase3/7-activation images were monitored for all HDACi and cell lines and analyzed to obtain the results in Figure 7.

**Figure 8:**
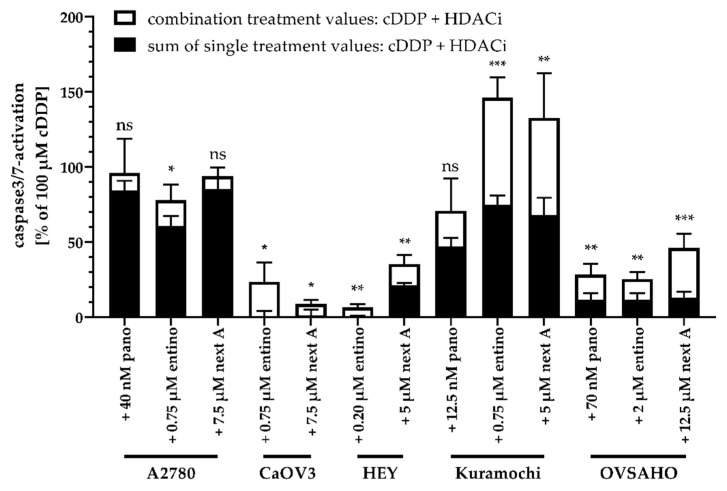
Synergistic effects of caspase3/7-activation upon combination treatment of HDACi and cisplatin. The sum of single treatment effects (HDACi, cisplatin) are shown in black bars. The extended white bars show the difference (superadditive (= synergistic) part) between the sum of single treatment effects and the effect of combination treatments. Results are shown for treatments significantly enhancing caspase3/7-activation (Figure 7). Before calculation, values were normalized to the effect of 100 µM cDDP and vehicle treated control was subtracted. Data shown are mean ± SD. Statistical analysis was performed using *t*-test. Levels of significance: ns (*p* > 0.05); * (*p* ≤ 0.05); ** (*p* ≤ 0.01); *** (*p* ≤ 0.001).

With exception of the panobinostat pretreatment in CaOV3 and HEY cells, every other experimental condition lead to an increase in caspase3/7-activation. Surprisingly, pan-HDAC-inhibition with panobinostat showed weaker effects than subtype-selective inhibition with entinostat or nexturastat A. In A2780, CaOV3, HEY, and Kuramochi cells, entinostat showed the strongest effect with exception of nexturastat A in OVSAHO. In order to determine whether the significant effects in caspase3/7-activation are synergistic, we compared the sum of the single treatment effects of HDACi and cisplatin (caspase3/7-activation) with the results from the combination treatments (Figure 8). Data were only analyzed if there was a significant increase in caspase3/7-activation in Figure 7.

Entinostat showed a significant synergistic effect on caspase3/7-activation in combination with cisplatin in every cell line. This synergism was mostly pronounced in Kuramochi cells as it reached the largest caspase3/7-activation. Caspase3/7-activation revealed a difference between the A2780 and the HGSOC cell lines. While in A2780 cells only entinostat triggered a synergistic effect (caspase3/7-activation) with cisplatin, both, entinostat and nexturastat A showed synergism with cisplatin in all HGSOC cell lines. However, it needs to be considered that nexturastat A was not HDAC6 selective in the concentrations used in this assay.

### 2.6. Alterations in Apoptosis-Related Gene Expression

**Figure 9:**
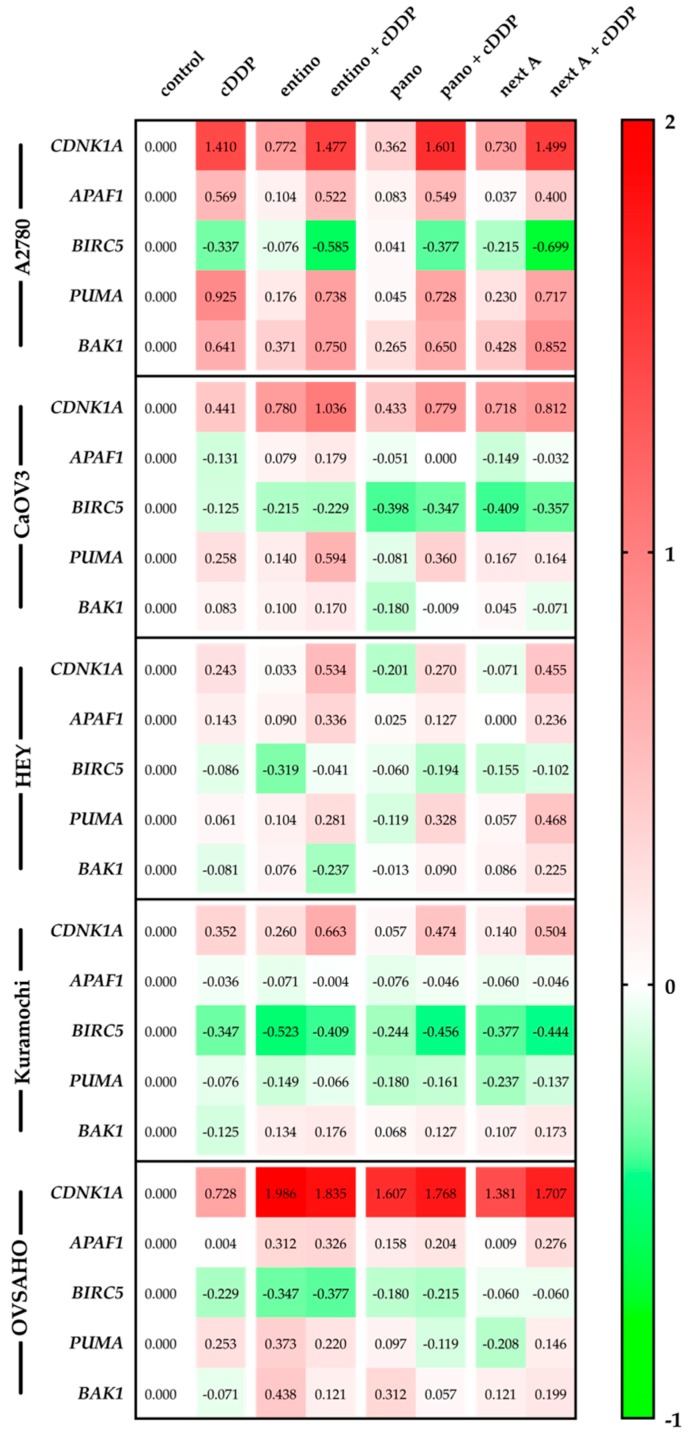
Gene expression data were obtained by RT-PCR and analyzed according to Vandesompele [40]. Cells were pretreated with concentrations of HDACi shown in Table 4 for 48 h followed by treatment with cisplatin for 24 h in an IC_50_ concentration. Data shown are normalized to endogenous control gene expression of *HPRT1* (hypoxanthine-guanine phosphoribosyltransferase), *TBP* (TATA binding protein), and *GUSB* (beta-glucuronidase) and rescaled to cell line specific control (72 h incubation with vehicle). Data are shown on a decadic logarithmic scale. Negative values marked in green show a lower expression compared to cell line specific control. Positive values marked in red show a higher expression compared to cell line specific control.

To get insight into the mechanism by which HDACi increase the potency of cisplatin in MTT assays, apoptosis induction and caspase activation, we performed RT-PCR-based gene expression analysis of apoptosis and cell cycle related genes. *CDNK1A* (cyclin-dependent kinase inhibitor 1; p21) is a p53-mediated regulator of cell cycle progression at the transition of G_1_- and S-phase and normally arrests the cell cycle in G_1_-phase after DNA damage [34]. As proapoptotic factors we investigated the expression of *APAF1* (apoptotic protease activating factor 1), *PUMA* (p53 upregulated modulator of apoptosis), and *BAK1* (Bcl-2 homologous antagonist killer) genes. APAF1 is a key factor for the induction of apoptosis, while it forms the apoptosome leading to the activation of the caspase cascade via activation of caspase 9 [35]. PUMA and BAK1 are both proapoptotic members of the Bcl-2 family. As a response to DNA damage, p53 is activated leading to the upregulation of PUMA itself [36]. It is discussed that PUMA interacts with antiapoptotic members of the Bcl-2 family (like Mcl-1, Bcl-2) and inhibits their inhibitory interaction with proapoptotic Bak (coding gene: *BAK1*) and Bax (also members of Bcl-2 family) resulting in a proapoptotic effect [37]. Upon induction, Bak leads to a loss of mitochondrial membrane potential and thus to cytochrome c release and apoptosis. It is also published that Bak forms an oligomeric pore and leads to the permeabilization of the outer membrane of mitochondria [38]. *BIRC5* (baculoviral inhibitor of apoptosis repeat-containing 5; survivin) was chosen as a representative antiapoptotic gene. It is a member of IAP (inhibitors of apoptosis) family and inhibits the activity of caspase 3/7 [39], which are downstream of caspase 9. BIRC5 leads to an inhibitory effect on apoptosis induction. Changes in apoptosis-related gene expression patterns are shown in Figure 9.

RT-PCR data showed varying levels of induction of *CDNK1A* (*p21*). While *p21* expression is strongly inducible by cisplatin and HDACi or combined treatment in A2780 and OVSAHO cells, the other cell lines showed a lower *p21* expression. Except for A2780 cells, the combination of HDACi and cisplatin showed a stronger induction of *p21* than cisplatin treatment alone. Interestingly, in OVSAHO cells, HDACi treatment alone had the same effect as the combination of HDACi and cisplatin on *p21* induction. In HEY and Kuramochi cell lines, HDACi only treatment had only moderate effects whereas the combination with cisplatin induces *p21*. In HEY cells, panobinostat and nexturastat A as single treatments led to slight downregulation of *p21*. Except for CaOV3 and Kuramochi cells, proapoptotic *APAF1* was upregulated by cisplatin and/or HDACi treatment. Mostly, combination treatments caused a higher upregulation than single treatment with HDACi. However, in A2780 cells, cisplatin single treatment was as effective in upregulating *APAF1* as combination treatments. The anti-apoptotic gene *survivin* (*BIRC5*) was downregulated as a result of the different treatment regimens. Mostly, the combination of HDACi with cisplatin caused a stronger downregulation of *survivin* gene than single treatments. Strongest downregulation of *survivin* was seen in A2780, CaOV3, and Kuramochi cells. Pro-apoptotic *PUMA* was upregulated under most treatment conditions except in Kuramochi cells. Whereas in A2780 cells combinations of HDACi and cisplatin were not superior to cisplatin alone, in CAOV3 and HEY cells combination treatments were more effective than cisplatin alone in upregulating *PUMA*. Pro-apoptotic *BAK1* expression was induced in A2780 cells upon cisplatin and HDACi treatment, however the combined treatment did only slightly increase *BAK1* expression. In the HGSOC cell lines, *BAK1* expression remained largely unaffected by cisplatin single treatment. HDACi mostly increased *BAK1* expression modestly. Combinations with cisplatin did not lead to additional *BAK1* expression in most cases—except for A2780 cells. As a general tendency, PCR studies showed an upregulation of *p21* and of proapoptotic genes *APAF1*, *PUMA*, *BAK1* as well as a downregulation of the antiapoptotic gene *survivin* (*BIRC5*) in ovarian cancer cell lines.

## 3. Discussion

Chemoresistance is the major problem in managing cancer. Among ovarian cancer, high grade serous ovarian carcinomas (HGSOC) have the lowest 5 year survival rate and do poorly respond to chemotherapy including platinum compounds [1,12]. Targeting epigenetic processes like DNA methylation or histone acetylation in tumors is a promising therapeutic strategy [20,21,22]. It is widely known that cancer cells exhibit hypoacetylation of histones (and other proteins) due to an overexpression of HDACs. Histone hypoacetylation leads to a more condensed heterochromatin structure of DNA with decreased expression of tumor suppressor genes and decreased access for DNA damaging agents like cisplatin [41]. HDACi are able to enhance the chemosensitivity of tumors for platinum-based drugs by normalizing the dysregulated process. Mechanisms behind this effect are among others alterations in the apoptosis-related gene expression pattern [42,43,44]. Studies with approved pan-HDACi like panobinostat and vorinostat (SAHA) explored their effects on gene expression [45,46], but there is still limited data available for cellular and transcriptional effects in solid tumors such as highly aggressive HGSOC. Furthermore, pan-HDACi have no selectivity towards an HDAC isoform or class and can cause severe side effects as known for panobinostat [47,48,49]. In our study, we compared the effects of one pan-HDACi and two isoform-selective HDACi on the cisplatin sensitivity of four HGSOC cell lines and—as control—one endometrioid adenocarcinoma type I (A2780). The aim was to explore if the use of a subtype-selective HDACi (entinostat, nexturastat A) has advantages over pan-HDACi (panobinostat).

The HDAC6-selective HDACi nexturastat A was efficacious in increasing cisplatin potency in the HGSOC and A2780 cells (Figure 3A,C,D, Table 6). However, the concentration needed to exert such effects was beyond HDAC6-selective concentrations. The K_i_ value of nexturastat A at HDAC6 was estimated as 0.03 µM whereas at HDAC2, the K_i_ was 1.25 µM. Even though this is a 42-fold selectivity for HDAC6, concentrations needed for nexturastat A to increase cisplatin potency were 5 µM and higher (Figure S2, Table 4, Table 5 and Table 6). In conclusion, effects seen with nexturastat A are in a concentration range where this compound was no longer selective but turned into a class I and class IIb HDACi. Thus, HDAC6 inhibition does not seem to play a major role in sensitization of HGSOC cells against cisplatin.

The pan-HDACi panobinostat was able to increase the cisplatin sensitivity significantly in two out of the four HGSOC cell lines (HEY, OVSAHO) and in A2780 cells. However, in HGSOC cells, panobinostat was less efficacious in increasing cisplatin sensitivity than the class I-HDACi entinostat as seen by lower shift factors for panobinostat (Table 6). In contrast to panobinostat, entinostat was able to significantly increase cisplatin sensitivity in all four HGSOC cell lines (Figure 1B,E, Figure S1A,E,G, Table 6). Since entinostat remains a selective class I HDACi in all concentrations used in this study, it can be concluded that class I HDAC inhibition is sufficient to explain the observed effects and that pan-HDAC inhibition is not only not required but possibly detrimental due to severe side effects associated with pan-HDACi [47,48,49].

HDACi-mediated increase in cisplatin potency was first estimated in cell viability assays (MTT) and required a preincubation of the HDACi prior to addition of cisplatin (Figure 3, Table 4 and Table 6). HDACi and cisplatin combinations were synergistic as shown by the Chou–Talalay analysis (Table 7). HDACi-mediated increase in cisplatin cytotoxicity (MTT) was shown to be associated with increased caspase3/7-activation and apoptosis induction (Figure 5, Figure 6, Figure 7 and Figure 8). Whereas combinatorial effects of cisplatin and nexturastat A or panobinostat, respectively, were not significant in all cell lines, the combinatorial effects of entinostat and cisplatin were always significant and synergistic (caspase3/7-activation, apoptosis induction) except for apoptosis induction in OVSAHO cells (Figure 5). This leaves the class I-HDACi entinostat as the most promising chemosensitizing HDACi in this study. Based on the presented results and the use of the caspase inhibitor QVD (not shown), it can be assumed that apoptosis induction by entinostat (or the other HDACi) and cisplatin treatment is caspase-3/7-driven in HGSOC cell lines.

The next question was what changes are induced by HDACi in HGSOC cells leading to chemosensitization against cisplatin? It is known that HDACi stimulate apoptosis in ovarian cancer cell lines by alteration of gene expression related to cell growth, cell cycle progression and apoptosis [50]. In hepatocellular carcinomas, proapoptotic *APAF1* is upregulated by HDACi [51]. Overexpression of *survivin* is known for most types of cancer and an association with high grade cancers and poor disease prognosis is well described [52]. It was shown in gastric cancer that overexpression of *BAK1* is related to induction of apoptosis [53]. High expression of *p21* may be a predictor for cisplatin sensitivity in ovarian carcinoma [54]. Proapoptotic *PUMA* is normally upregulated by p53 [55]. These literature results prompted us to analyze the gene expression profile of the five apoptosis and survival-related genes *APAF1*, *survivin*, *BAK1*, *p21*, and *PUMA* by PCR in all ovarian cancer cells used in this study under single and combination treatments of HDACi with cisplatin (Figure 9). As a general summary, we found an upregulation of *p21* and proapoptotic genes and a downregulation of *survivin* upon HDACi treatment. However, variability within the different cell lines was rather large. Whereas *survivin* was downregulated in all cell lines upon entinostat treatment, *APAF1*, *PUMA*, and *BAK1* expression were upregulated in all cell lines except Kuramochi cells in which only *BAK1* was upregulated upon entinostat treatment. The same holds true for *PUMA* expression which is regulated by p53 [55]. Since p53-mutations are quite often observed in HGSOC cancer cell lines (e.g., CaOV3, Kuramochi, and OVSAHO [23,25,26]), it is not surprising that *PUMA* is less upregulated in these cells compared to p53 wt A2780 cells. Although literature describes a direct link between *p21* expression and cisplatin sensitivity [54], we could not establish a quantitative link in our cell lines (Figure 9, Table 6). Still, the gene expression analysis upon HDACi treatment of five apoptosis and survival-related genes can explain facilitated apoptosis upon cisplatin treatment in HGSOC and A2780 cells and is in full agreement with literature data on gene expression of proapoptotic genes presented above.

In conclusion, HDACi are able to increase the sensitivity of HGSOC cancer cell lines towards cisplatin. The class I-selective HDACi entinostat turned out superior in increasing cisplatin potency than pan-HDAC inhibition in cell viability assays (MTT), apoptosis induction (subG1), and caspase3/7-activation. Further, entinostat is synergistic with cisplatin in all cell lines in MTT and caspase activation assays. Combination indices estimated according to Chou–Talalay were <0.9, indicating synergism. Mechanistically, HDACi induce an upregulation of cell cycle arrest and pro-apoptotic genes (*CDNK1A*, *APAF1*, *PUMA*, *BAK1*) and repression of prosurvival genes such as *survivin* which is in accordance with literature data for different types of cancer [56,57]. In conclusion, the combination of entinostat and cisplatin is synergistic in HGSOC and could be an effective strategy for the clinical treatment of this aggressive ovarian cancer subtype. Furthermore, studies on HDACi-induced gene expression changes (Figure 9) may lead to predictive biomarkers for cisplatin sensitivity in HGSOC.

## 4. Materials and Methods

### 4.1. Reagents

Cisplatin was purchased from Sigma (München, Germany) and dissolved in 0.9% sodium chloride solution, propidium iodide (PI) was purchased from PromoKine (Heidelberg, Germany). Stock solutions (10 mM) of entinostat, nexturastat A, and panobinostat (Selleckchem, Houston, Texas, USA) were prepared with DMSO and diluted to the desired concentrations with the appropriate medium. All other reagents were supplied by PAN Biotech (Aidenbach, Germany) unless otherwise stated.

### 4.2. Cell Lines and Cell Culture

The human ovarian carcinoma cell lines A2780 and HEY were obtained from European Collection of Cell Cultures (ECACC, Salisbury, UK). The cell lines Kuramochi and OVSAHO were obtained from Japanese Collection of Research Bioresources Cell Bank (JCRB Cell Bank, Osaka, Japan). CaOV3 cell line was obtained from ATCC/LGC Standards GmbH (Wesel, Germany). All cell lines were grown at 37 °C under humidified air supplemented with 5% CO_2_ in RPMI 1640 (A2780, HEY, Kuramochi, OVSAHO) or DMEM (CaOV3) containing 10% heat inactivated fetal calf serum, 120 IU/mL penicillin, and 120 µg/mL streptomycin. The cells were grown to 80% confluency before being used in further assays.

### 4.3. MTT Cell Viability Assay

The rate of cell-survival under the action of test substances was evaluated by an improved MTT assay as previously described [20,31]. To investigate the effect of entinostat, panobinostat, and nexturastat A on cisplatin induced cytotoxicity, compounds were added 48 h before cisplatin administration. After 72 h, the cytotoxic effect was determined by MTT assay as described above (density of cell seeding for this incubation scheme: A2780 3000 cells/well (c/w), CaOV3 3000 c/w, HEY 3500 c/w, Kuramochi 4500 c/w, and OVSAHO 10,000 c/w), and shift factors were calculated by dividing the IC_50_ value of cisplatin alone by the IC_50_ value of the drug combinations. Incubation was ended after 72 h and cell survival was determined by addition of MTT (Serva, Heidelberg, Germany) solution (5 mg/mL in phosphate buffered saline). The formazan precipitate was dissolved in DMSO (VWR, Langenfeld, Germany). Absorbance was measured at 544 nm and 690 nm in a FLUOstar microplate-reader (BMG LabTech, Offenburg, Germany).

### 4.4. Whole-Cell HDAC Inhibition Assay

The cellular HDAC assay was based on an assay published by Heltweg and Jung [58], Ciossek et al. [59], and Bonfils et al. [60] with minor modifications as described in [20]. Briefly, human cancer cell lines A2780, CaOV3, HEY, Kuramochi, and OVSAHO were seeded in 96-well tissue culture plates (Corning, Kaiserslautern, Germany) at a density of 15,000 c/w, 15,000 c/w, 17,500 c/w, 22,500 c/w, and 50,000 c/w in a total volume of 90 µL of culture medium. After 24 h, cells were incubated for 18 h with increasing concentrations of test compounds. The reaction was started by adding 10 µL of 3 mM Boc-Lys(Ac)-AMC (Bachem, Bubendorf, Switzerland) to reach final concentration of 0.3 mM [61]. The cells were incubated with the Boc-Lys(Ac)-AMC for 3 h under cell culture conditions. After this incubation, 100 µL/well stop solution (25 mM Tris-HCl (pH 8), 137 mM NaCl, 2.7 mM KCl, 1 mM MgCl2, 1 % NP40, 2.0 mg/mL Trypsin, 10 µM Vorinostat) was added and the reaction was developed for 3 h under cell culture conditions. Fluorescence intensity was measured at excitation of 320 nm and emission of 520 mm in a NOVOstar microplate reader (BMG LabTech, Offenburg, Germany).

### 4.5. Combination Experiments

For the investigation of the effect of HDACi on cisplatin induced cytotoxicity, the compounds were added 48 h before cisplatin administration. After 72 h, the cytotoxic effect was determined with a MTT cell viability assay. Calcusyn software 2.1 (Biosoft, Cambridge, UK) was used to calculate the combination index (CI) as a quantitative measure of the degree of drug interaction.

### 4.6. Enzyme HDAC Inhibition Assay

All human recombinant enzymes were purchased from Reaction Biology Corp. (Malvern, PA, USA). The HDAC activity assay HDAC2 (cat nr. KDA-21-277), HDAC4 (cat nr. KDA-21-279), HDAC6 (cat nr. KDA-21-213), and HDAC8 (cat nr. KDA-21-481) was performed in 96-well-plates (Corning, Kaiserslautern, Germany). Briefly 20 ng of HDAC2/8, 17.5 ng of HDAC6 and 2 ng of HDAC4 per reaction were used. Recombinant enzymes were diluted in assay buffer (50 mM Tris-HCl, pH 8.0, 137 mM NaCl, 2.7 mM KCl, 1 mM MgCl_2_, and 1 mg/mL BSA). After a 5 min incubation step the reaction was started with 10 µL of 300 µM (HDAC2), 150 µM (HDAC6) Boc-Lys(Ac)-AMC (Bachem, Bubendorf, Switzerland) or 100 µM (HDAC4), 60 µM (HDAC8) Boc-Lys-(TFa)-AMC (Bachem, Bubendorf, Switzerland). The reaction was stopped after 90 min by adding 100 µL stop solution (16 mg/mL trypsin, 2 µM panobinostat for HDAC2/6/8, 2µM CHDI0039 (kindly provided by the CHDI Foundation Inc., New York, USA) for HDAC4 in 50 mM Tris-HCl, pH 8.0, and 100 mM NaCl. 15 min after the addition of the stop solution the fluorescence intensity was measured at excitation of 355 nm and emission of 460 nm in a NOVOstar microplate reader (BMG LabTech, Offenburg, Germany).

### 4.7. Measurement of Apoptotic Nuclei

A2780, CaOV3, HEY, Kuramochi, and OVSAHO cells were seeded at a density of 40,000 c/w, 50,000 c/w, 40,000 c/w, 60,000 c/w, and 100,000 c/w in 24-well plates (Sarstedt, Nürnbrecht, Germany). Cells were treated with entinostat, panobinostat, or nexturastat A and cisplatin alone or in combination for the indicated time points. Supernatant was removed after a centrifugation step and the cells were lysed in 500 µL hypotonic lysis buffer (0.1% sodium citrate, 0.1% Triton X-100, 100 µg/mL PI) at 4 °C in the dark overnight. The percentage of apoptotic nuclei with DNA content in sub-G1 was analyzed by flow cytometry using the CyFlow instrument (Partec, Norderstedt, Germany).

### 4.8. Caspase 3/7 Activation Assay

Compound-induced activation of caspases 3 and 7 was analyzed using the CellEvent Caspase-3/7 green detection reagent (Thermo Scientific, Wesel, Germany) according to the manufacturer’s instructions. Briefly, A2780, CaOV3, HEY, Kuramochi, and OVSAHO cells were seeded in 96-well-plates (Corning, Kaiserslautern, Germany) at a density of 4000 c/w, 7500 c/w, 7500 c/w, 4500 c/w, and 10000 c/w. Cells were treated with entinostat, panobinostat, or nexturastat A 48 h prior to the addition cisplatin and another incubation period of 24 h. Then, medium was removed and 50 µL of CellEvent Caspase 3/7 green detection reagent (2 µM in PBS supplemented with 5% heat inactivated FBS) was added. Cells were incubated for 30 min at 37 °C in a humidified incubator before imaging by using the Thermo Fisher ArrayScan XTI high content screening (HCS) system with a 10× magnification (Thermo Scientific). Hoechst 33342 was used for nuclei staining. The pan caspase inhibitor QVD was used in a concentration of 20 µM diluted in the appropriate medium and incubated 30 min prior to compound addition.

### 4.9. Immunoblotting

Cells were treated with indicated concentrations of entinostat, panobinostat, or nexturastat A for 48h prior to cisplatin (24 h) or 72 h entinostat, panobinostat, or nexturastat A without cisplatin, or vehicle for 72 h. Cell pellets were dissolved with RIPA buffer (50 mM Tris-HCl pH8.0, 1% Triton X-100, 0.5% sodium deoxycholate, 0.1% SDS, 150 mM sodium chloride, 2 mM EDTA, supplemented with protease and phosphatase inhibitors (Pierce™ protease and phosphatase inhibitor mini tablets, Thermo Scientific, Wesel, Germany)) and clarified by centrifugation. Equal amounts of total protein (20 µg unless otherwise stated) were resolved by SDS-PAGE and transferred to polyvinylidene fluoride membranes (Merck Millipore, Darmstadt, Germany). PageRuler Prestained Protein Ladder, 10 to 180 kDa (Thermo Scientific, Wesel, Germany) was used as protein molecular weight marker. Blots were incubated with primary antibodies against acetylated α-tubulin (Cat. No. sc-23950), α-tubulin (Cat. No. sc-8035) (Santa Cruz Biotechnology, Heidelberg, Germany), histone H3 (Cat. No. MAB9448), acetyl histone H3 (Lys24, Cat. No. NBP2-54615), HDAC1 (Cat. No. NB100-56340), HDAC2 (Cat. No. MAB7679), HDAC3 (Cat. No. NB100-1669), HDAC4 (Cat. No. AF6205), HDAC5 (Cat. No. NBP2-22152), HDAC6 (Cat. No. NB100-56343), HDAC10 (Cat. No. NB100-91801), and HDAC11 (Cat. No. NBP2-16789) (Biotechne, Wiesbaden, Germany). Immunoreactive proteins were visualized using luminol reagent (Santa Cruz Biotechnology, Heidelberg, Germany) with an Intas Imager (Intas, Göttingen, Germany).

### 4.10. RT-PCR

Cells were treated with indicated concentrations of entinostat, panobinostat, or nexturastat a for 48 h prior to cisplatin (24 h) or 72 h entinostat, panobinostat, or nexturastat A without cisplatin, or cell culture medium for 72 h. RNA was isolated using RNeasy Mini Kit (Qiagen, Hilden, Germany). Afterwards transcription to cDNA was performed with High Capacity cDNA Reverse Transcription Kit (Thermo Scientific, Wesel, Germany). RT-PCR was performed with GoTaq qPCR Master Mix (Promega, USA) in a CFX96 Real-Time System (BIO-RAD, Hercules, California, USA). Relative changes in gene expression were normalized to endogenous control genes *GUSB* (beta-glucuronidase), *TBP* (TATA binding protein), and *HPRT1* (hypoxanthine-guanine phosphoribosyltransferase) by Vandesompele method [40] and if indicated normalized to vehicle treated control. Primers (Sigma Aldrich, Steinheim, Germany) with an efficacy between 80% and 115% were used (Table 8). They were designed by Primer-BLAST (NIH, Bethesda, Maryland, USA) [62] and efficacy was determined with a template isolated from HeLa cells.

### 4.11. Data Analysis

Concentration-effect curves were constructed with Prism 7.0 (GraphPad, San Diego, CA, USA) by fitting the pooled data of at least three experiments performed in triplicate to the four-parameter logistic equation. Statistical analysis was performed using *t*-test or one-way ANOVA. To analyze the synergistic effects of apoptosis induction and caspase3/7-activation, the values of the single treatments were summed up and the standard deviation calculated. This value was compared with the actual measured value of the combination treatment using *t*-test.

## Figures and Tables

**Table 1:** IC_50_ and pIC_50_ for cisplatin after a 72h incubation.

Cell Line	IC_50_ [µM]	pIC_50_ ± SEM
**A2780**	11.0	4.96 ± 0.01
**CaOV3**	1.44	5.84 ± 0.01
**HEY**	5.25	5.28 ± 0.01
**Kuramochi**	4.68	5.33 ± 0.02
**OVSAHO**	4.83	5.32 ± 0.01

Data shown are the mean of pooled data from at least three experiments, each carried out in triplicate.

**Table 2:** Cytotoxic activity of entinostat, panobinostat, and nexturastat A.

Cell Line	HDACi					
	Entinostat		Panobinostat		Nexturastat A	
	IC_50_ [nM]	pIC_50_ ± SEM	IC_50_ [nM]	pIC_50_ ± SEM	IC_50_ [nM]	pIC_50_ ± SEM
**A2780**	606	6.22 ± 0.03	15.3	7.82 ± 0.02	7778	5.11 ± 0.09
**CaOV3**	1146	5.94 ± 0.03	7.62	8.12 ± 0.03	5291	5.28 ± 0.03
**HEY**	251	6.60 ± 0.02	2.68	8.57 ± 0.02	1724	5.76 ± 0.03
**Kuramochi**	485	6.31 ± 0.03	11.2	7.95 ± 0.02	5302	5.28 ± 0.02
**OVSAHO**	1828	5.74 ± 0.02	42.4	7.37 ± 0.05	16,218	4.79 ± 0.02

Cell viability was measured by MTT assay after 48 h preincubation followed by an additional 72 h incubation. Data shown are the mean of pooled data from at least three experiments each carried out in triplicates.

**Table 3:** HDAC inhibitory activity of entinostat, panobinostat, and nexturastat A.

Cell Line	HDACi					
	Entinostat		Panobinostat		Nexturastat A	
	IC_50_ [nM]	pIC_50_ ± SEM	IC_50_ [nM]	pIC_50_ ± SEM	IC_50_ [nM]	pIC_50_ ± SEM
**A2780**	313	6.50 ± 0.04	12.1	7.91 ± 0.06	3633	5.44 ± 0.04
**CaOV3**	333	6.48 ± 0.03	7.88	8.10 ± 0.05	3500	5.46 ± 0.03
**HEY**	219	6.60 ± 0.04	13.6	7.87 ± 0.03	3874	5.41 ± 0.03
**Kuramochi**	339	6.47 ± 0.04	9.87	8.01 ± 0.07	3733	5.43 ± 0.03
**OVSAHO**	326	6.49 ± 0.02	23.1	7.64 ± 0.04	5249	5.28 ± 0.02

Data shown are the mean of pooled data from at least three experiments each carried out in triplicates.

**Table 4:** HDACi concentrations used for combination treatment.

Cell Line	Entinostat [nM]	Panobinostat [nM]	Nexturastat A [nM]
**A2780**	750	40.0	7500
**CaOV3**	750	15.0	7500
**HEY**	200	20.0	5000
**Kuramochi**	750	12.5	5000
**OVSAHO**	2000	70.0	12,500

**Table 5:** IC_50_ and inhibitory constants (K_i_) of nexturastat A on HDAC2, 4, 6, and 8.

	HDAC2		HDAC4		HDAC6		HDAC8	
Compd.	IC_50_ ± SD [µM]	K_i_ [µM]	IC_50_ ± SD [µM]	K_i_ [µM]	IC_50_ ± SD [µM]	K_i_ [µM]	IC_50_ ± SD [µM]	K_i_ [µM]
**Nexturastat A**	1.99 ± 0.25	1.25	12.0 ± 1.69	7.57	0.05 ± 0.01	0.03	22.6 ± 2.55	12.8

Data shown are the mean ± SD of pooled data from at least three experiments each carried out in triplicates.

**Table 6:** Effect of HDACi pretreatment on cisplatin-induced cytotoxicity (MTT assay).

Cell Line	Cisplatin	+ 48 h HDACi Pretreatment					
		Entinostat		Panobinostat		Nexturastat A	
	IC_50_	IC_50_	SF	IC_50_	SF	IC_50_	SF
**A2780**	11.0	4.99	2.2 (***)	2.58	5.6 (***)	1.44	7.6 (***)
**CaOV3**	1.44	0.72	2.0 (*)	0.74	2.0 (ns)	1.33	1.1 (ns)
**HEY**	5.25	1.39	3.8 (***)	2.75	1.9 (*)	1.28	4.1 (***)
**Kuramochi**	4.68	3.28	1.4 (*)	5.14	< 1 (ns)	3.22	1.5 (**)
**OVSAHO**	4.83	1.02	4.7 (***)	3.01	1.6 (**)	1.43	3.4 (***)

Data shown are the mean of pooled data from at least three experiments, each carried out in triplicate. Shift-factors (SF) were calculated by dividing the IC_50_-values without and with HDACi-preincubation. pIC_50_ and SEM are shown in supplemental Table S1. Statistical analysis was performed using *t*-test. Levels of significance: ns (*p* > 0.05); * (*p* ≤ 0.05); ** (*p* ≤ 0.01); *** (*p* ≤ 0.001).

**Table 7:** Synergism studies (CI-values) between HDACi entinostat, panobinostat, or nexturastat A and cisplatin.

	Cisplatin [µM]	Entinostat [µM]				Panobinostat [nM]				Nexturastat A [µM]			
		0.10	0.25	0.50	0.75	10	20	30	40	1.25	2.50	5.00	7.50
**A2780**	**0.50**	*	0.40	0.36	0.38	0.21	0.10	0.15	0.15	*	0.32	0.24	0.28
	**1.00**	0.64	0.38	0.35	0.39	0.25	0.12	0.17	0.18	*	0.36	0.28	0.32
	**2.00**	0.83	0.43	0.34	0.40	0.35	0.16	0.22	0.21	0.97	0.47	0.30	0.33
	**4.00**	0.73	0.44	0.34	0.38	0.35	0.21	0.27	0.25	>1.1	0.57	0.38	0.39
	**6.00**	0.59	0.35	0.32	0.37	0.29	0.23	0.26	0.26	0.83	0.52	0.37	0.40
		**0.25**	**0.50**	**0.75**	**1.00**	**-**	**-**	**-**	**-**	**-**	**-**	**-**	**-**
**CaOV3**	**0.20**	*	0.66	0.71	0.42	◦	◦	◦	◦	◦	◦	◦	◦
	**0.40**	*	0.56	0.59	0.33	◦	◦	◦	◦	◦	◦	◦	◦
	**0.60**	0.56	0.41	0.49	0.28	◦	◦	◦	◦	◦	◦	◦	◦
	**0.80**	0.33	0.30	0.31	0.23	◦	◦	◦	◦	◦	◦	◦	◦
	**1.00**	0.21	0.20	0.21	0.16	◦	◦	◦	◦	◦	◦	◦	◦
		**0.10**	**0.15**	**0.20**	**0.25**	**5.0**	**10.0**	**15.0**	**20.0**	**1.25**	**2.50**	**3.75**	**5.00**
**HEY**	**0.32**	*	*	*	0.27	*	*	0.19	0.14	*	*	0.32	0.27
	**0.50**	*	*	*	0.26	*	*	0.21	0.15	*	*	0.33	0.29
	**1.00**	*	*	0.31	0.25	*	*	0.25	0.16	*	*	0.36	0.27
	**2.00**	*	0.37	0.29	0.24	*	0.41	0.25	0.17	*	0.63	0.33	0.26
	**4.00**	0.35	0.28	0.24	0.23	0.51	0.33	0.28	0.20	0.62	0.42	0.29	0.27
		**0.25**	**0.50**	**0.75**	**1.00**	**-**	**-**	**-**	**-**	**1.25**	**2.50**	**3.75**	**5.00**
**Kuramochi**	**1.00**	0.43	0.42	0.51	0.62	◦	◦	◦	◦	*	0.73	0.66	0.66
	**2.00**	0.42	0.43	0.49	0.60	◦	◦	◦	◦	*	0.78	0.74	0.72
	**3.00**	0.46	0.44	0.48	0.57	◦	◦	◦	◦	*	0.80	0.77	0.75
	**4.00**	0.44	0.42	0.44	0.53	◦	◦	◦	◦	0.84	0.79	0.77	0.74
	**5.00**	0.45	0.43	0.47	0.52	◦	◦	◦	◦	0.78	0.78	0.74	0.70
		**0.50**	**1.00**	**1.50**	**2.00**	**40**	**50**	**60**	**70**	**5.00**	**7.50**	**10.0**	**12.5**
**OVSAHO**	**1.00**	0.50	0.39	0.41	0.44	0.36	0.33	0.27	0.26	*	0.82	0.84	0.78
	**2.00**	0.41	0.29	0.34	0.37	0.25	0.23	0.21	0.21	0.87	0.77	0.75	0.67
	**3.00**	0.42	0.28	0.32	0.36	0.24	0.22	0.21	0.21	0.80	0.76	0.65	0.60
	**4.00**	0.39	0.28	0.31	0.35	0.23	0.22	0.21	0.21	0.72	0.69	0.61	0.57
	**5.00**	0.39	0.29	0.33	0.36	0.24	0.23	0.22	0.22	0.64	0.59	0.53	0.53

Data shown are combination indices (CI) calculated using CompuSyn 1.0 based on the Chou–Talalay method. CI > 1.1 indicates antagonism, CI = 1 indicates an additive effect, and CI < 0.9 indicates synergism. * means fraction affected is less than 0.20. Values are the mean of two experiments. SD is < 10 % of the mean. Combinations marked with ◦ were not investigated because they did not show significant shift factors in combination treatment as can be seen in Table 6.

**Table 8:** Primer sequences for RT-PCR.

Gene	Primer Forward	Primer Reverse	Efficacy [%]
*HPRT1*	CCTGGCGTCGTGATTAGTGA	CGAGCAAGACGTTCAGTCCT	93.6
*TBP*	GTGACCCAGCATCACTGTTTC	GAGCATCTCCAGCACACTCT	86.9
*GUSB*	ACCTCCAAGTATCCCAAGGGT	GTCTTGCTCCACGCTGGT	83.1
*HDAC1*	TGCAAAGAAGTCCGAGGCAT	ACCCTCTGGTGATACTTTAGCA	84.9
*HDAC2*	AATGGAAATATATAGGCCCC	GTTATCTGGTCTTATTGACCG	96.4
*HDAC3*	GGCAACTTCCACTACGGAGC	GCATATTGGTGGGGCTGACT	97.2
*HDAC4*	TTGGATGTCACAGACTCCGC	CCTTCTCGTGCCACAAGTCT	80.8
*HDAC5*	GGAGAGCTCAAGAATGGATTTGC	CTGCTGTAGGAGTTTTGCG	97.2
*HDAC6*	CTGGCGGAGTGGAAGAACC	TCTGCCTACTTCTTCGCTGC	104
*HDAC7*	TCTCGTGAGCTAAAGAATGG	CTGTTGAATGATCTGCATGG	96.5
*HDAC8*	CCACCTTCCACACTGATGCT	GCTGGGCAGTCATAACCTAGC	97.7
*HDAC9*	TGTAGCTGGTGGAGTTCCCT	CTCTGAGGCAAAGGTGCAGA	103
*HDAC10*	TGGCCTTTGAGTTTGACCCT	CCGATGGCTGAGTCAAATCCT	97.3
*HDAC11*	CGGAAAATGGGGCAAAGTGA	CAACAGCAAAGGACCACTTG	100
*CDNK1A (p21)*	TGCCGAAGTCAGTTCCTTGT	GTTCTGACATGGCGCCTCC	94.7
*BIRC5 (Survivin)*	TGAGAACGAGCCAGACTTGG	TGTTCCTCTATGGGGTCGTCA	108
*APAF1*	AGTGGAATAACTTCGTATGTAAGGA	AAACAACTGGCCTCTGTGGT	98.7
*BAK1*	TCATCGGGGACGACATCAAC	CAAACAGGCTGGTGGCAATC	111
*PUMA*	GAGCGGCGGAGACAAGAG	TAAGGGCAGGAGTCCCATGA	94.7

## Supplementary Materials

Supplementary materials can be found at https://www.mdpi.com/1422-0067/20/12/3052/s1.

## Abbreviations

HDAC	histone deacetylase
HDACi	histone deacetylase inhibitor
entino	entinostat
cDDP	*cis*-diamminedichloridoplatinum(II) (cisplatin)
pano	panobinostat
next A	nexturastat A
BIRC5, survivin	baculoviral inhibitor of apoptosis repeat-containing 5
PUMA	p53 upregulated modulator of apoptosis
BAK/Bak	Bcl-2 homologous antagonist killer
CDNK1A, p21	cyclin-dependent kinase inhibitor 1
APAF1	apoptotic protease activating factor 1
IAP	inhibitors of apoptosis
HPRT1	hypoxanthine-guanine phosphoribosyltransferase
GUSB	beta-glucuronidase
TBP	TATA binding protein
HGSOC	high grade serous ovarian cancer
MTT	3-(4,5-Dimethylthiazole-2-yl)-2,5-diphenyltetrazoliumbromide
CI	combination index
RT-PCR	reverse transcriptase polymerase chain reaction
