# Supplementary material for: Class I-Histone Deacetylase (HDAC) Inhibition is Superior to pan-HDAC Inhibition in Modulating Cisplatin Potency in High Grade Serous Ovarian Cancer Cell Lines
Source: Int J Mol Sci. 2019 Jun 22;20(12):3052. doi: 10.3390/ijms20123052 (PMC6627993; doi:10.3390/ijms20123052)
Supplement: Supplementary file 1 [file ijms-20-03052-s001.pdf]

Table S1. Effect of HDACi pretreatment on cisplatin-induced cytotoxicity.

cell line	cisplatin	+ 48 h HDACi pretreatment					
		entinostat		panobinostat		nexturastat A	
	IC_{50} [pIC_{50} ± SEM]	IC_{50} [pIC_{50} ± SEM]	SF	IC_{50} [pIC_{50} ± SEM]	SF	IC_{50} [pIC_{50} ± SEM]	SF
**A2780**	11.0 [4.96 ± 0.01]	4.99 [5.30 ± 0.05]	2.2 ***	2.58 [5.59 ± 0.04]	5.6 ***	1.44 [5.84 ± 0.06]	7.6 ***
**CaOV3**	1.44 [5.84 ± 0.01]	0.72 [6.14 ± 0.05]	2.0 *	0.74 [6.13 ± 0.05]	2.0 ns	1.33 [5.88 ± 0.04]	1.1 ns
**HEY**	5.25 [5.28 ± 0.01]	1.39 [5.89 ± 0.03]	3.8 ***	2.75 [5.56 ± 0.04]	1.9 *	1.28 [5.89 ± 0.04]	4.1 ***
**Kuramochi**	4.68 [5.33 ± 0.02]	3.28 [5.48 ± 0.02]	1.4 *	5.14 [5.29 ± 0.04]	< 1 ns	3.22 [5.49 ± 0.02]	1.5 **
**OVSAHO**	4.83 [5.32 ± 0.01]	1.02 [5.99 ± 0.03]	4.7 ***	3.01 [5.52 ± 0.02]	1.6 **	1.43 [5.84 ± 0.05]	3.4 ***

Data shown are the mean of pooled data from at least three experiments each carried out in triplicates. Shift-factors (SF) were calculated by dividing the IC_{50}-values without and with HDACi-preincubation. Levels of significance: ns ($p > 0.05$); * ($p \le 0.05$); ** ($p \le 0.01$); *** ($p \le 0.001$).

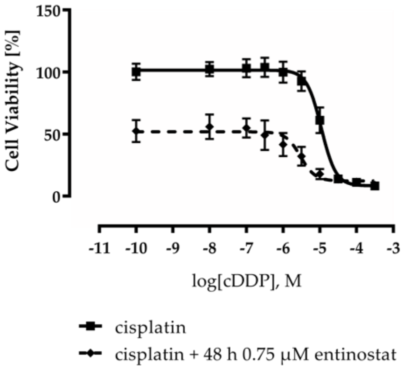
Cell Viability [%]
150
100
50
0
-11 -10 -9 -8 -7 -6 -5 -4 -3
log[cDDP], M
cisplatin
cisplatin + 48 h 0.75 μM entinostat

(a)

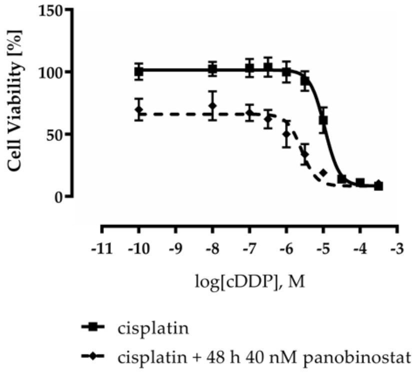
Cell Viability [%]
150
100
50
0
-11 -10 -9 -8 -7 -6 -5 -4 -3
log[cDDP], M
cisplatin
cisplatin + 48 h 40 nM panobinostat

(b)

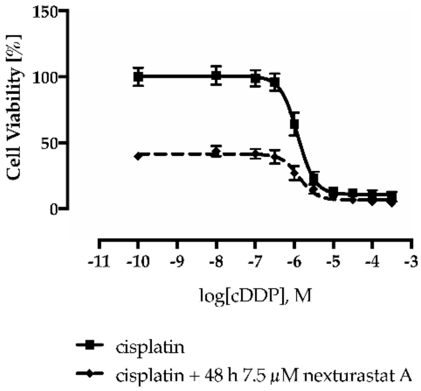
Cell Viability [%]
150
100
50
0
-11 -10 -9 -8 -7 -6 -5 -4 -3
log[cDDP], M
cisplatin
cisplatin + 48 h 7.5 μM nexturastat A

(c)

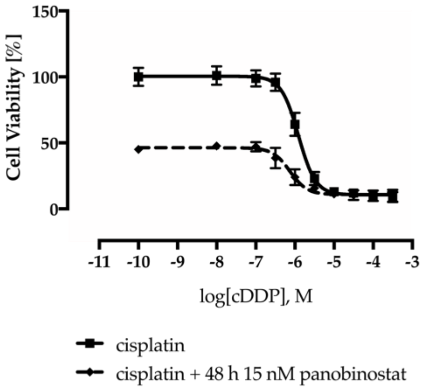
Cell Viability [%]
150
100
50
0
-11 -10 -9 -8 -7 -6 -5 -4 -3
log[cDDP], M
cisplatin
cisplatin + 48 h 15 nM panobinostat

(d)

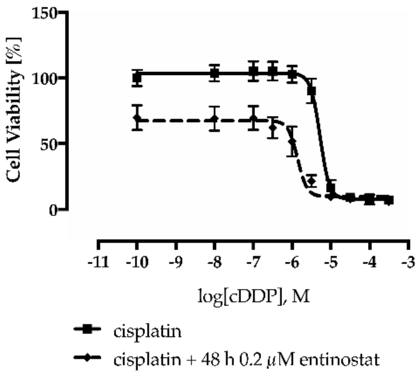
Cell Viability [%]
150
100
50
0
-11 -10 -9 -8 -7 -6 -5 -4 -3
log[cDDP], M
cisplatin
cisplatin + 48 h 0.2 μM entinostat

(e)

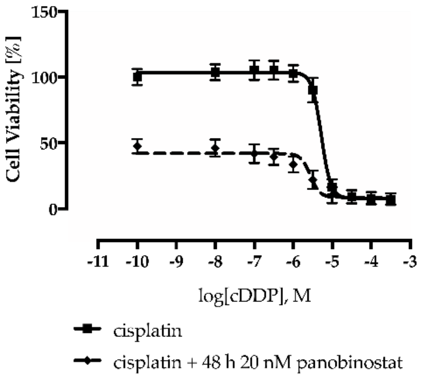
Cell Viability [%]
150
100
50
0
-11 -10 -9 -8 -7 -6 -5 -4 -3
log[cDDP], M
cisplatin
cisplatin + 48 h 20 nM panobinostat

(f)

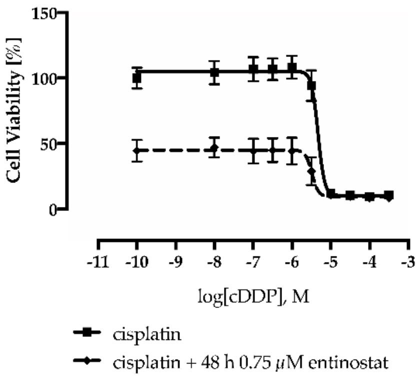
Cell Viability [%]
150
100
50
0
-11 -10 -9 -8 -7 -6 -5 -4 -3
log[cDDP], M
cisplatin
cisplatin + 48 h 0.75 μM entinostat

(g)

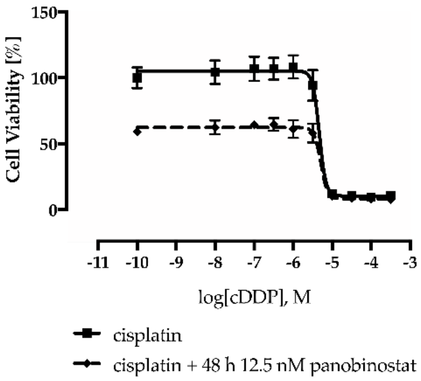
Cell Viability [%]
150
100
50
0
-11 -10 -9 -8 -7 -6 -5 -4 -3
log[cDDP], M
cisplatin
cisplatin + 48 h 12.5 nM panobinostat

(h)

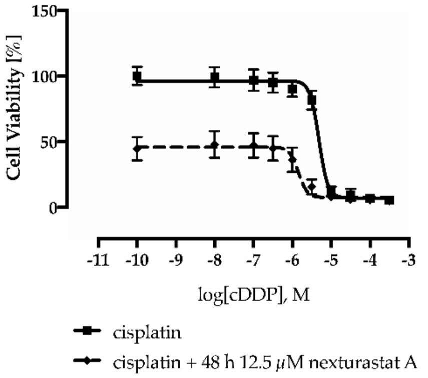

(i)

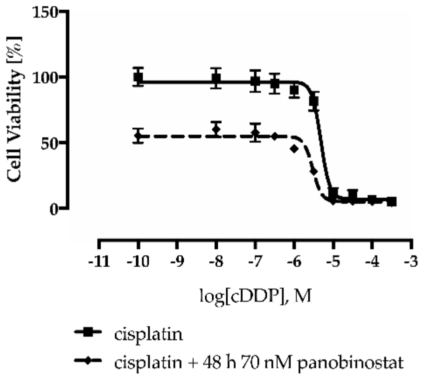

(j)

Figure S1. HDACi pretreatment enhance the cytotoxic effects of cisplatin. A2780 (a,b), CaOV3 (c,d), HEY (e,f), Kuramochi (g,h), and OVSAHO (i,j) were pretreated with the indicated HDACi 48 h prior to cisplatin (cDDP) administration. After another 72 h, IC_{50}-values were determined by MTT-assay. Data shown are normalized to vehicle control and pooled of at least two experiments each carried out in triplicates.

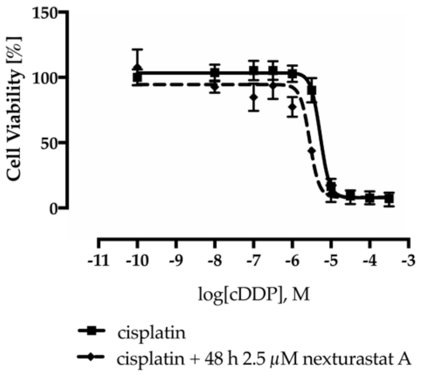

(a)

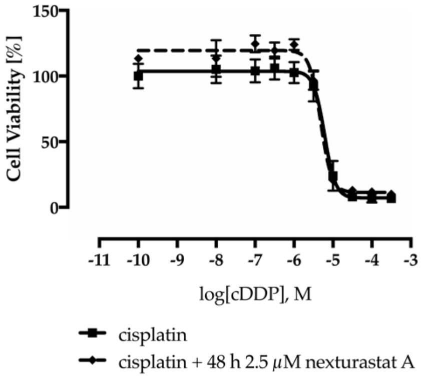

(b)

Figure S2. Pretreatment with nexturastat A in low concentrations did not enhance the cytotoxic effects of cisplatin. HEY (a) and CaOV3 (b) cells were pretreated with the indicated HDACi 48 h prior to cisplatin (cDDP) administration. After another 72 h, IC50-values were determined by MTT-Assay. Data shown are normalized to vehicle control and pooled of two (a) or one (b) experiment each carried out in triplicates.

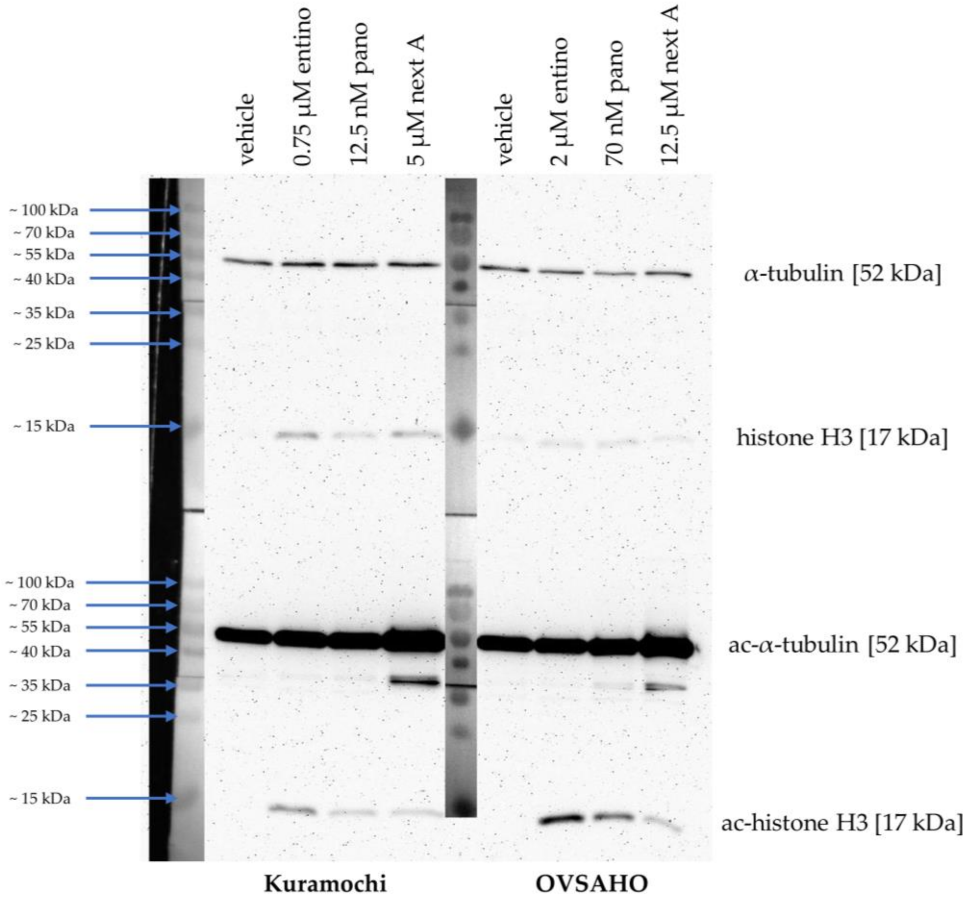

Figure S3. Effect of HDACi on acetylation level of α-tubulin and histone H3. Representative immunoblot analysis of histone H3, ac-histone H3, α-tubulin, and ac-α-tubulin in Kuramochi and OVSAHO cells. Cells were treated with the indicated concentrations of HDACi. Control cells were incubated with vehicle. Protein molecular weight marker is indicated on the left side.
